# Bioavailability and In Vivo Antioxidant Activity of a Standardized Polyphenol Mixture Extracted from Brown Propolis

**DOI:** 10.3390/ijms20051250

**Published:** 2019-03-12

**Authors:** Valeria Curti, Vincenzo Zaccaria, Arold Jorel Tsetegho Sokeng, Marco Dacrema, Irene Masiello, Anna Mascaro, Giuseppe D’Antona, Maria Daglia

**Affiliations:** 1 Department of Drug Sciences, Medicinal Chemistry and Pharmaceutical Technology Section, Pavia University, Viale Taramelli 12, 27100 Pavia, Italy; valeria.curti86@hotmail.it (V.C.); vincenzo.zaccaria01@universitadipavia.it (V.Z.); aroldjorel.tseteghosokeng01@universitadipavia.it (A.J.T.S.); irene.masiello01@universitadipavia.it (I.M.); 2 Department of Pharmacy, University of Napoli Federico II, Via D. Montesano 49, 80131 Naples, Italy; marco.dacrema01@universitadipavia.it; 3 Department of Public Health, Experimental and Forensic Medicine and CRIAMS-Sport Medicine Centre Voghera, Pavia University, Viale Foscolo 12, 27058 Voghera, Italy; anna.mascaro01@universitadipavia.it (A.M.); gdantona@unipv.it (G.D.A.)

**Keywords:** brown propolis, standardized polyphenol mixture, bioavailability, in vivo antioxidant activity, superoxide dismutase

## Abstract

Several lines of evidence demonstrate the antioxidant, anti-inflammatory and antimicrobial activities of propolis, mostly ascribed to its polyphenol content. However, little is known regarding the bioavailability of propolis in acute and prolonged settings of oral administration. In this study, we first determined the content of the main polyphenols in a brown propolis extract obtained using a patented extraction method (Multi Dinamic Extraction—M.E.D.) by RP-HPLC-UV-PDA-MSn analysis, followed by the bioavailability of galangin and chrysin, the most abundant polyphenols in the mixture (7.8% and 7.5% respectively), following acute (single bolus of 500 mg/kg containing about 3.65 mg of the polyphenol mixture) and prolonged (100, 250 and 500 mg/kg body for 30 days) oral administration in 30 male 8 weeks old C57BL/6 wild-type mice. In the acute setting, blood was taken at 30 s and 5, 10, 15, 20, 25, 30, 45, 60 and 120 min following the oral bolus. In the prolonged setting, blood samples were obtained after 10, 20 or 30 days of administration. At the end of treatment, expression of antioxidant enzymes (superoxyde dismutase, SOD-1; catalase, CAT; glutathione peroxidase, GSS) was evaluated in liver tissue. Following both acute and prolonged administration, neither galangin nor chrysin were detectable in the plasma of mice, whereas the glucuronide metabolite of galangine was detectable 5 min after acute administration. At the end of the prolonged treatment SOD-1 was found to have increased significantly, unlike CAT and GSS. Overall, these data suggest that oral administration of whole brown propolis extract is followed by rapid absorption and metabolization of galangin followed by adaptations of the antioxidant first line defense system.

## 1. Introduction

Propolis is a resinous product which bees (especially *Apis mellifera*) process from numerous botanical sources [1,2,3]. European brown propolis and Brazilian green propolis are two of the many types that can be distinguished based on plant origin, possessing different chemical compositions and, consequently, biological activities. This complex mixture typically consists of resins (40%–60%), beeswax (20%–40%), essential oils (10%), pollen (5%) and mineral elements (2%). More than 300 organic compounds, which represent about 5% of the total, have been identified, including vitamins, chalcones, dihydrochalcones, polyphenols (flavonoids and non-flavonoids) and terpenoids [4,5,6,7].

Hundreds of in vitro studies have confirmed the healthy properties of propolis, which can be mainly ascribed to antioxidant, anti-inflammatory and antimicrobial activities mostly mediated by polyphenolic compounds and terpenes [8,9,10,11]. As far as the antioxidant activity is concerned, propolis was demonstrated to show free-radical scavenging capacities and protective effects against lipid peroxidation [12]. Moreover, brown propolis was found to increase miR-27a-3p, thus negatively affecting the abundance of the transcription factor NFE2L2, generally associated with oxidative stress. In addition, brown propolis down-regulated miR-17-3p, responsible for the regulation of some mitochondrial antioxidant enzymes, revealing its protective activity against oxidative damage exerted through an epigenetic mechanism of action [13].

To our knowledge, the number of studies describing the bioavailability of propolis are limited, this bioavailability representing the amount of propolis and/or its components able to be absorbed into a living system and to reach the sites in which they may exert their biological effects through systemic circulation [14]. This parameter can be affected by many factors (i.e., the food matrix, possible interactions with other compounds, environment, chemical structure and concentration) and it is therefore difficult to evaluate [15]. In the literature, human studies on propolis bioavailability dated back to the ‘80s. Until now, propolis shows low bioavailability, which in turn, reduces propolis therapeutic effects. In 1987, propolis was tested in a clinical trial by Hausen et al. at the dose of 3600 mg/day. The concentrations of glucuronide and sulfate metabolites in blood, determined through HPLC method, were at nanomolar level. To improve the detection of propolis metabolites, in the same study, the oral daily intake was increased to 8000 mg, which resulted to be an excessive dose for the patients [16]. More recently, Gardana et al. (2007) demonstrated significant increase in plasma polyphenols within few hours (5 h) after the ingestion of a propolis standardized extract corresponding to 125 mg of flavonoids. These levels decreased significantly after 8 h and were no longer detectable after 24 h [17]. The difficulty in determining the bioavailability of propolis has prompted researchers to focus their studies on single components of propolis, instead of the combined substance, though this approach has some limitations due to the absence of the propolis matrix which in practice would exert a crucial role in the absorption of this product [18]. For instance, Konishi et al. [19,20] showed that the intestinal adsorption efficiency of artepillin C, one of the main phenolic acids in green propolis, was lower than its in vitro transcellular passive diffusion through a Caco-2 monolayer, underlining the necessity of in vivo investigations to deepen our understanding of propolis bioavailability and its biological activities. In particular, artepillin C was demonstrated to be less efficiently absorbed than p-coumaric acid due to the involvement of the monocarboxylic acid transporter (MCT) [19,21], revealing that the permeation of polyphenols depends on several factors including the possible activity of membrane channels. This molecule was also described as being susceptible to hepatic elimination, thus reducing its bioavailability [19]. The adsorption kinetics and bioavailability of other pure phenolic acids have also been defined, such as p-coumaric, gallic, caffeic and rosmarinic acids [20,22,23]. Despite the great amount of available data on the biological activities of propolis, in vivo data are very scarce. Despite this, antioxidant capacity remains the most studied property [8,10,12]. For instance, as far as the in vivo effects of propolis are concerned, its oral administration to rats (60 mg/kg for 14 days) was shown to reduce the toxic effects of chemotherapeutic drugs modulating the glutathione/glutathione-S-transferase turnover, suggesting the capture of reactive metabolites [24]. Additionally, propolis extracts were demonstrated to protect the gastric mucosa from oxidative stress, showing an anti-ulcer action due in particular to coumaric and cinnamic acids [25], as well as spasmolytic activity and protective effects from ethanol-induced lesions [26]. An overview of the in vivo studies of propolis or propolis polyphenols, while limited, seems to confirm some of its well known in vitro properties, such as its ROS scavenging activity and protective effects against lipid peroxidation and superoxide dismutase (SOD-1) activity [27].

Considering the importance of bioavailability in being able to accurately evaluate the in vivo antioxidant capacity of propolis (which, in turn, can play a role against oxidative stress linked to the development of most chronic diseases) [28,29], the aim of this study was to evaluate the bioavailability of a standardized polyphenol mixture obtained from brown propolis, whose composition is reproducible independently from propolis geographical origins and seasonal and climatic conditions because of its particular extraction procedure, administered under acute and prolonged treatments. The in vivo antioxidant activity was also analyzed following the prolonged treatment evaluating the concentration of three antioxidant enzymes (SOD-1; catalase, CAT; glutathione synthase, GSS). European propolis was chosen because of its higher antioxidant activity compared to green propolis, as reported by Zaccaria et al. (2017) [13]. 

## 2. Results

### 2.1. Analysis of Propolis Polyphenolic Content

Prior to conducting the in vivo bioavailability evaluation of M.E.D. extract of brown propolis, the more abundant polyphenols occurring in the propolis sample were assessed by RP-HPLC-UV-PDA-MSn analysis. The concentration of total polyphenols was found to be 7.21 ± 0.15 mg/g, with galangin as the most abundant polyphenol (7.8% ± 0.5%), followed by chrysin (7.5% ± 0.5%), pinocembrin (5.0% ± 0.3%) and others occurring at concentrations less than 2% (Table 1).

The analysis of the polyphenolic content of propolis identified galangin and chrysin as the two most representative compounds of the propolis sample, which were chosen as markers for the characterization of the bioavailability of brown propolis following acute or prolonged treatments.

#### 2.1.1. Acute Treatment

In order to evaluate the dosage for the acute and prolonged treatments of mice, the human daily intake of polyphenols from propolis was calculated, considering the following data: (1) the average total polyphenol intake in humans, which is estimated as between 600 to 1800 mg/day, as reported by Zamora-Ros et al. (2016) [30]; (2) the estimated average daily intake of propolis (for instance through food supplements), which ranges from 0.5 to 1.0 g; (3) the concentration of polyphenols occurring in the tested propolis sample (7.21 mg/g). Therefore, the human daily intake of polyphenols from propolis ranges from 3.61 to 7.21 mg, which represents 0.2%–1.2% of the total intake of polyphenols coming from all other dietary foods and beverages. The propolis dosages for animal treatments were calculated using the polyphenol human equivalent dose (HED), which is about 17 mg/kg for a 70 kg person. Then, the animal dose was obtained by normalizing to the body surface area through the following formula: ANIMAL DOSE = (HED × Km_Human_)/Km_mouse_, where HED is the human equivalent dose, Km is the correction factor, with Km_Human_ is 37 and Km_mouse_ is 3, corresponding to a daily dose of 210 mg/kg polyphenol. Thus, considering the highest daily intake of polyphenols derived from propolis as 1.2%, we calculated the doses of polyphenols for the experimental animals as approximately 2.5 mg/kg corresponding to about 350 mg/kg of the tested propolis. For the acute treatment, the dosage was raised to 500 mg/kg (corresponding to 140% of polyphenols commonly consumed with propolis).

**Figure 1:**
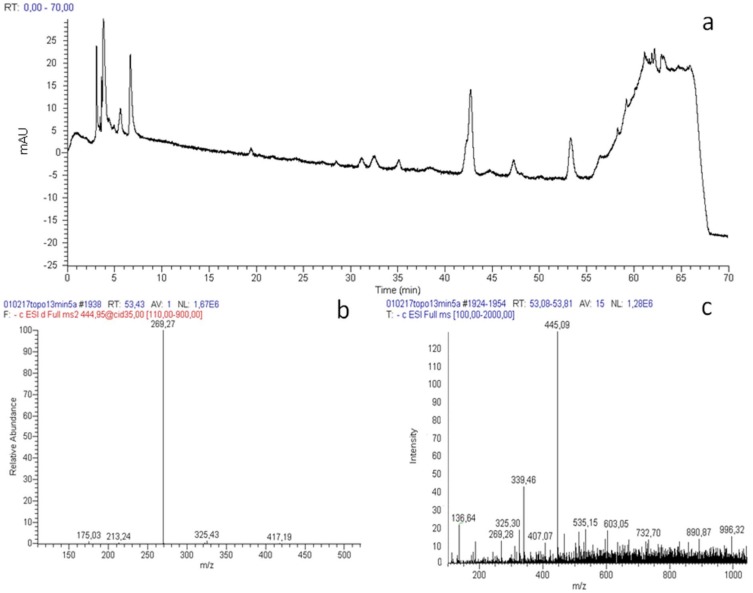
(**a**) The chromatogram of the blood sample collected after 5 min of acute treatment on mouse 13 is shown. (**b**) The MS spectrum is relative to the peak with a retention time of 53.34, which is galangin–glucuronide. (**c**) This MS/MS spectrum describes the fragmentation of galangin–glucuronide with m/z 445: the parent ion, detected in negative ionization mode, produces the fragments at m/z 269, 157, 305 and 361.

**Figure 2:**
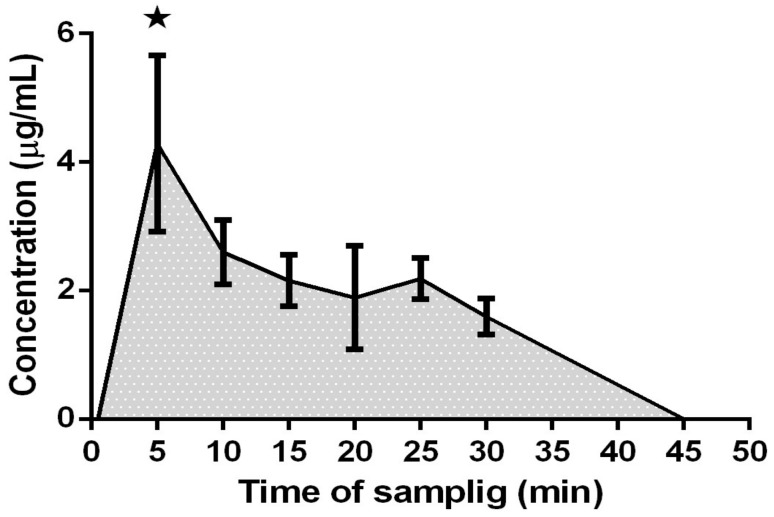
Galangine–glucuronide concentration in plasma samples collected at different times is shown: after 5 min this metabolite reaches its highest concentration in plasma; then, the concentration maintains a plateau; finally, after 45 min from the treatment, it is no longer detectable. Asterisk indicates significantly different from the other time points (*p* < 0.05).

As far as the acute treatment is concerned, blood samples were taken at 30 sec, 5, 10, 15, 20, 25, 30, 45, 60 and 120 min. The analysis of plasma samples by RP-HPLC-UV-PDA-MSn showed that neither galangin nor chrysin were present in blood after the acute treatment. Thus, we next verified the presence of galangin and chrysin metabolites. Only galangin–glucuronide (retention time (t_R_) = 53.34 min; Figure 1a) was identified after 5 min of acute treatment on the basis of its MS and MS-MS spectra. In fact, the parent ion at m/z 445, detected in negative ionization mode, produces the fragments at m/z 269, 157, 305 and 361 (Figure 1b,c), in accordance with data reported in literature [31]. Galangin–glucuronide concentration, expressed as galangin equivalents, was found to be 4.29 μg/mL. The changes in galangin–glucuronide concentration at different times following acute administration is reported in Figure 2: after 5 min this metabolite increased, reaching a plateau between 10 and 25 min; then the concentration progressively decreased until 45 min, after which galangin–glucuronide was no longer detectable. The measured area under the curve (AUC) was 80.33 ± 6.2 (μg. h)/mL.

#### 2.1.2. Prolonged Treatment

For the prolonged treatment, mice were divided into four groups which were fed with different propolis concentrations for 30 days, with the exception of the control group: these doses being 100 mg/kg (corresponding to 30% of polyphenols commonly consumed with propolis), 250 mg/kg (corresponding to 70% of polyphenols commonly consumed with propolis) and 500 mg/kg (corresponding to 140% of polyphenols commonly consumed with propolis). Analysis conducted on plasma samples withdrawn one hour after last propolis intake following this prolonged administration revealed the absence of any propolis components. Similarly, galangin, chrysin and their metabolites, such as galangin–glucuronide, were investigated by means of RP-HPLC-UV-PDA-MSn, and the results showed that they had not accumulated in mouse liver (data not shown).

### 2.2. In Vivo Evaluation of Antioxidant Enzymes

The results showing that galangin could be absorbed, but promptly metabolized to the glucuronide derivatives, prompted us to evaluate the systemic effects induced by propolis treatment and, in particular, the antioxidant activity in terms of a possible increase in the endogenous defenses.

**Figure 3:**
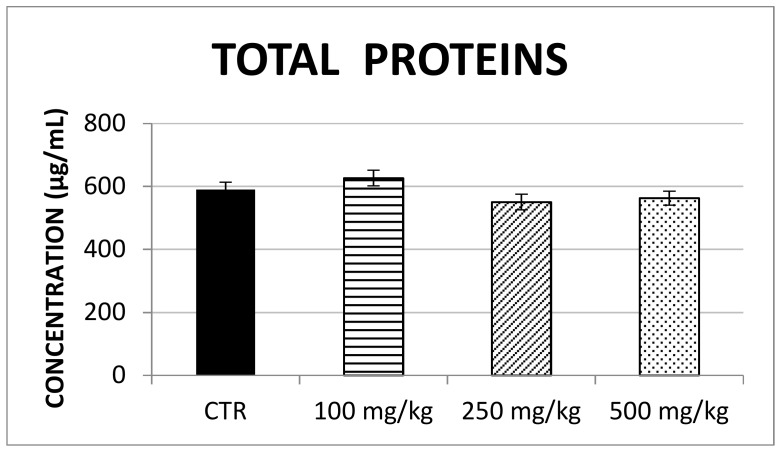
The concentration of soluble liver proteins calculated using the BCA assay is reported in control and treated groups. CTR = control.

First, the concentration of soluble proteins in mouse liver was calculated for control and treated mice (Figure 3). An aliquot of 25 mg of mouse liver was homogenized in 5 mL of PBS (5 mg/mL). The concentration of soluble proteins was expressed as µg of soluble proteins/mL of liver homogenate, referring to the BSA calibration curve to normalize the quantification of the antioxidant enzymes (i.e., SOD-1, CAT and GSS). Thus, the antioxidant enzymes were determined by the ELISA technique, using the specific calibration curve. The average and standard deviation of enzyme concentration (expressed as pg of enzyme/µg of soluble proteins) were calculated per animal and then per group of mice treated with the same propolis dosage. 

**Figure 4:**
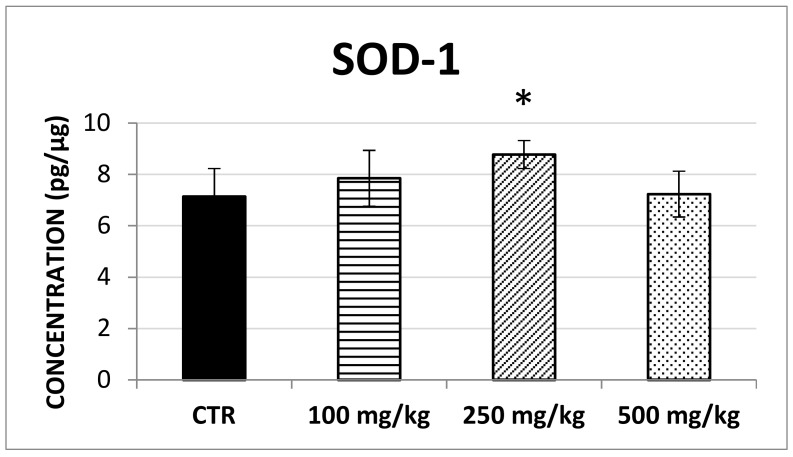
The average SOD-1 concentration in the control group and in mice treated with different dosages of propolis expressed in pg/mg of soluble liver protein. A significant difference could be detected after the prolonged treatment, with 250 mg/kg of propolis extract compared to the control (* means a *p* = 0.0106).

**Figure 5:**
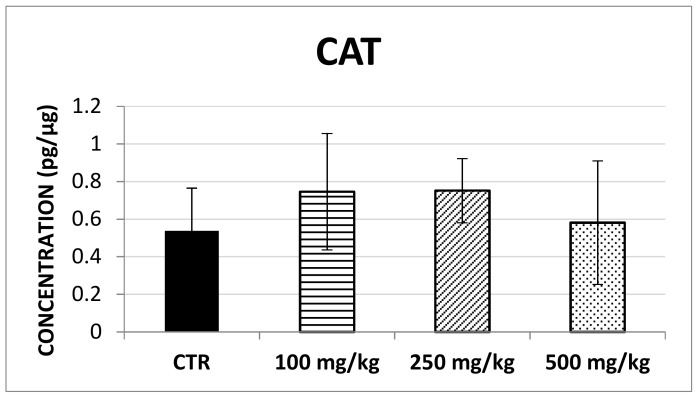
This histogram illustrates CAT concentration in control and treated groups. No significant differences could be found between groups.

**Figure 6:**
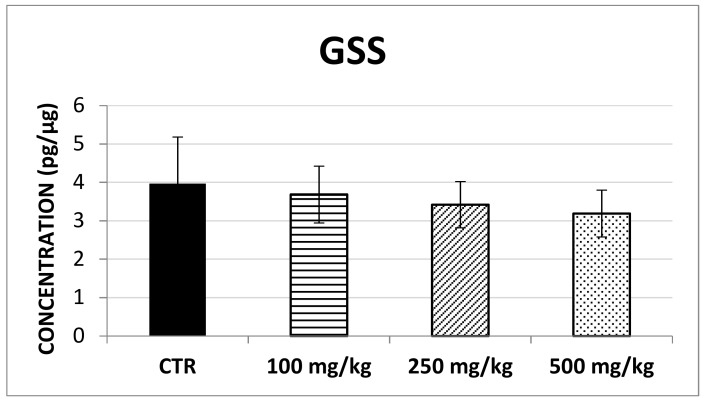
This histogram illustrates GSS concentration in control and treated groups. No significant differences could be found between groups.

The results showed that SOD-1 concentration significantly increased following brown propolis treatment at 250 mg/kg (F (3,60) = 4.07; *p* < 0.05) though this was not found for the 100 mg/kg and 500 mg/kg treatment (Figure 4). Similarly, no changes in CAT concentration (Figure 5) and GSS concentration (Figure 6) were detected after any of the propolis treatments.

## 3. Discussion

We analyzed the bioavailability and antioxidant activity of a standardized polyphenol mixture obtained from brown propolis in mice, applying acute and prolonged treatments with the following three main results: (1) galangin could be absorbed, but was immediately converted to its glucuronide derivative; (2) neither galangin nor its derivatives were accumulated in the liver; (3) propolis exerted in vivo antioxidant effects, increasing the concentration of SOD-1, which represents one of the fundamental endogenous antioxidant defenses.

### 3.1. Bioavailability

In order to characterize the in vivo antioxidant properties of propolis and its consequent impact on health, we first aimed to study its bioavailability. In particular, using the mouse animal model, we investigated the capacity of propolis polyphenols to reach the bloodstream after acute administration. While galangin and chrysin were found to have similar concentrations in the propolis sample, only galangin was detectable in blood, and in its glucuronated form, whereas chrysin and its metabolites were not detectable. These results showed that galangin could be adsorbed and rapidly glucuronidated following oral administration, and also suggested that galangin, chrysin and their derivatives have a short bioavailability in mice.

These results partially agree with those Chen et al. (2015) [31] obtained in orally-treated rats with galangin at a concentration of 10 mg/kg body weight: in fact, the authors identified galangin glucuronide, also finding free galangin unlike our study. This discrepancy could be due to the much higher dosage used compared to our experimental conditions (about 70 times higher). In fact, in our experimental conditions, mice fed with 500 mg of bolus/kg body weight ingested 3.65 mg of polyphenols/kg. With galangin representing 7.8% of total polyphenols in the tested propolis sample, the intake in mice was about 285 µg of galangin/kg body weight, corresponding to a dosage of galangin of 142 µg/kg in rats (km_rat_ = 6) instead of 10mg/kg used by Chen et al.

For a complete analysis of the bioavailability of galangin or chrysin, we studied their levels in blood following a prolonged treatment, but neither the two compounds nor their derivatives were detected. Moreover, metabolites were not detected in the liver, showing that no accumulation occurred at this site. In addition to confirming the absorption and short bioavailability of propolis polyphenols taken into account, the results from our study represent the first evidence to our knowledge of the lack of propolis polyphenol accumulation in the liver, which prevents potential harmful effects at this level.

### 3.2. In Vivo Antioxidant Effect

In spite of its low bioavailability, galangin absorption and metabolization in healthy mice prompted us to verify the in vivo antioxidant effects induced by the administration of propolis polyphenolic standard mixture. Thus, we investigated the liver concentration of SOD-1, CAT and GSS, three key enzymes involved in the endogeneous antioxidant defenses. Only SOD-1, which represents the first and the most important antioxidant line of defence, acting on superoxide anions, was found to increase significantly (percentage increase = 23%) after a prolonged treatment of 250 mg/kg of the polyphenolic standardized mixture. Our results showed a comparable trend to those observed by Jasprica et al. (2006) [27]. Nevertheless, some differences in these studies have to be highlighted, in fact they underline a significant increase of extracellular SOD (EC-SOD) in men after a daily intake of propolis extract for 30 days. In particular, in this study the authors demonstrated a statistically significant increase in plasma EC-SOD activity (+20.9%) and decreased plasma malondialdehyde (MDA) concentration (−23.2%) at the end of prolonged (30 days) propolis administration in men unlike women. These results demonstrated that dietary intervention with propolis reduced free-radical-induced lipid peroxidation thus highlighting its potential role in prevention of oxidative stress-related diseases, such as degenerative, cardiovascular, and cancer, in humans. Importantly, in this experimental condition the decrease in MDA concentration reached its maximum after the initial 15 days of propolis supplementation. This finding suggested gender dependence and transitory effect of propolis consumption on lipid peroxidation and showed that long-term propolis usage under the described experimental conditions does not necessary exhibit any beneficial effect. Furthermore, in our study GSS enzyme concentration did not show any significant difference in treated mouse liver in comparison with the concentration determined in the untreated mouse liver. These results are not in agreement with the results obtained by Zhao et al. that in the presence of a different type of propolis (Brazilian green propolis at the dose of 900 mg/day for 18 weeks) found an increase in serum levels of GSH [32]. Overall, considering that this antioxidant enzyme is generally elevated following a certain degree of oxidative stress, we could first hypothesize that a hormetic level of stress following propolis administration could promote the transitory and dose dependent potentiation of the first line of cellular defense. This potential mechanism agrees with the observation that SOD-1 upregulation may be promoted through the activation of transcriptional factors, including Nuclear Factor-KappaB (NF-κB) and CCAAT-Enhancer-Binding Protein (C/EBP) signaling pathways which may be modulated by polyphenols, at least in healthy subjects [33,34].

## 4. Materials and Methods

### 4.1. Materials

The following materials were used in the experiments: a standardized polyphenol mixture extracted from brown propolis (brown propolis M.E.D. extract, B Natural S.r.l, Corbetta, Italy); Synergi Fusion RP-18 column (150 × 4.6 mm, 5 μm) equipped with a Hypersil Gold C18 precolumn (10 × 2.1 mm, 5 μm) (Phenomenex, Torrance, CA, USA); formic acid and methanol (Sigma Aldrich, Missouri, MO, USA); phosphate buffer saline solution (1× PBS) (EuroClone, Milan, Italy); Halt Protease Inhibitor (Thermo Fisher Scientific, Waltham, MA, USA); Microplate BCA Protein Assay Kit – Reducing Agent Compatible (Thermo Fisher Scientific, Waltham, MA, USA); and an Enzyme-Linked Immunosorbent Assay (ELISA) Kit (Cloud-Clone Corp., Huston, TX, USA).

### 4.2. Determination of the Main Polyphenols in Brown Propolis M.E.D. Extract

Brown propolis dry extract (obtained using a patented extraction method called Multi Dinamic Extraction—M.E.D.) was prepared as reported in Zaccaria et al. (2017) [13]. In brief, the MED^®^ method was used to eliminate inactive resins, and obtain a propolis complex concentrate, rich in polyphenols both in free and glycosylated forms. The process included an initial aqueous extraction followed by three hydro-alcoholic extractions using different alcoholic degrees and temperatures, from 4 to 36 h, with a fixed 1:1 solvent/propolis residue ratio. The water and hydro-alcoholic extracts were combined and concentrated through the evaporation of ethanol. The residue was mixed with glycerin and water and then filtered to obtain a hydro glycerine extract. 

The most abundant polyphenols in the sample were quantified by a RP-HPLC-PDA-Esi-MSn method [13,35] using a Thermo Finnigan Surveyor Plus HPLC equipped with a quaternary pump, a Surveyor UV-Vis diode array detector, and a LCQ Advantage ion trap mass spectrometer (Thermo Fisher Scientific, Waltham, MA, USA); XCalibur Software (2.0 SR2 Thermo Fisher Scientific, Waltham, MA, USA) was used for data analysis. In brief, compound separation was obtained with an analytical Synergi Fusion RP-18 column (150 × 4.6 mm, 5 µm), equipped with a Hypersil Gold C18 precolumn (10 × 2.1 mm, 5 µm), all produced by Phenomenex (Torrance, CA, USA). The mobile phase used was acidified water (0.1% formic acid) (eluent A) and methanol (eluent B) as reported by Zaccaria et al. [13]. The run time was 110 min in total, including the reconditioning of the column. The volume of injection was set to 5 µL. The flow rate was 1.00 mL/min, and the temperatures of the autosampler and column were kept at 4 and 33 °C, respectively. Chromatograms were registered at five different wavelengths (254, 280, 330, 370 and 395 nm).

### 4.3. Animal Model and Diet

A total of 29 adult male mice C57BL/6 (Charles River, Massachusetts, MA, USA) 8 weeks old (average weight about 20 g), were first acutely orally administered (gavage) with a dose of 500 mg/kg propolis containing about 3.65 mg of the polyphenol mixture dissolved in 0.4 mL of ethanol, under anesthesia (intraperitoneal administration of 0.024 mL/g avertin) to minimize animal suffering. Each mouse was subjects to two retro-orbital bleedings according to Sharma et al. (2014) [36] at different time intervals as described in Table 2. During procedures mice were maintained at constant core temperature by using a thermoregolatory home-made device (maintained rectal temperature of 36 ± 1 °C). Following one week from the acute procedure, 16 out of 29 mice were then subjected to the prolonged administration trial, being divided in four groups of 4 mice each undergoing a prolonged treatment (each day for 30 days by gavage with 0.2 mL/day of polyphenols containing ethanol solution). One group was held as control and was daily sham treated with equal volume of ethanol by gavage, and the other three were feed with propolis at the following concentrations: 100, 250 and 500 mg/kg body weight/day. During treatment mice were fed with standard diet (D12450B Charles River) not containing polyphenols. Blood drawings were obtained after 10, 20 or 30 days and at the end of the prolonged treatment, one hour after the last administration, mice were culled by cervical dislocation to collect their liver. In each post-intake time plasma samples were analyzed: 5 times for 5 min, 15 min, 20 min, 60 min, 120 min after bolus; 6 times for 10 min and 25 min after bolus; 7 times for 45 min after bolus and 10 times for 30 min after bolus Animal procedures were approved by the Institutional Animal Ethic Committee of the University of Pavia (11.07.2016) with Ministry of Health authorization project identification code 1086/2016-PR. 

### 4.4. Analysis of Propolis Bioavailability

#### 4.4.1. Plasma Sample Preparation

Mouse plasma was analyzed after both acute and prolonged treatments, aiming to detect any propolis metabolites in systemic circulation. Plasma was first separated from the corpuscular parts, and 100 μL were mixed with 300 μL of methanol to be centrifuged at 13,000 rpm for 10 min at 4 °C for protein precipitation. The supernatant was separated from the precipitate and supernatant methanol evaporated with nitrogen at low temperature. The samples were then reconstituted with 100 μL of methanol and centrifuged as before to collect the supernatants for HPLC-UV-PDA-MSn analysis.

#### 4.4.2. Extraction of Galangin and Its Metabolites from Liver

Following prolonged treatment, immediately after culling, mouse liver was collected to evaluate the possible accumulation of propolis metabolites. An amount of 500 mg of liver was frozen at −80 °C and then ground with 3 mL of methanol acidified with 0.1% formic acid. The samples were sonicated for 30 min and centrifuged at 13,000 rpm for 10 min at 4 °C. The supernatant methanol was separated from the precipitate. Methanol was evaporated with nitrogen at low temperature, protected from light, and the residues were reconstituted in 100 μL of methanol and centrifuged again for HPLC-UV-PDA-MSn analysis.

#### 4.4.3. RP-HPLC-PDA-ESI-MSn

Both plasma and liver samples were analyzed by a RP-HPLC-PDA-ESI-MSn method [30]. Chromatographic analysis was performed using a Thermo Finnigan Surveyor Plus HPLC system (Waltham, MA, USA) equipped with a quaternary pump, a Surveyor UV-Vis diode array detector (Waltham, MA, USA) and a LCQ Advantage ion trap mass spectrometer (Waltham, MA, USA). Separation of propolis compounds was conducted using a Synergi Fusion RP-18 column with a Hypersil Gold C18 pre-column. The mobile phases were 0.1% formic acid in water (eluent A) and 100% methanol (eluent B), with the gradient elution method described in Table 3. The flow rate was set at 0.500 mL/min. The temperature of the autosampler was maintained at 4 °C with the column at 30 °C; the injection volume was 5 μL. Chromatograms were registered at 207, 266 and 395 nm; UV-Vis spectra were also registered in the range of 200–800 nm. The data were analyzed by Xcalibur Software (Waltham, MA, USA) and using a calibration curve prepared with solutions of standard galangin at different concentrations (1, 2, 4, 8, 12 and 15 ng/mL).

### 4.5. Antioxidant Enzyme Quantification

Concentration of antioxidant enzymes (SOD-1, CAT and GSS) was calculated to reveal possible in vivo effects of the chosen standardized pholyphenol mixture on endogenous antioxidant defenses.

#### 4.5.1. Liver Sample Preparation

Once defrosted, liver samples were washed with PBS, and 25 mg of each sample were homogenized with a dounce homogenator, adding 5 mL of PBS with 1% protease inhibitor, then being kept on ice. The homogenized samples were centrifuged at 13,000 rpm for 10 min at 4 °C to remove tissue debris. The supernatants were used for quantification of the soluble fraction of the proteins occurring in the liver homogenate by a BCA protein assay kit Fisher Scientific Italia (Rodano, Milan, Italy) and, then, for quantification of the antioxidant enzymes by ELISA kits (Segrate, Milan, Italy).

#### 4.5.2. Bicinchoninic Acid Assay

A total of 9 μL of each homogenized sample was loaded into a multi-well plate with 4 μL of reducing agent: the plate was mixed for 1 min and then incubated for 15 min at 37 °C. Finally, 260 μL of working reagent were added to each well, stirred for 1 min and incubated first for 30 min at 37 °C and then for 5 min at room temperature. The absorbance was measured at 562 nm to calculate the soluble protein content. Standard solutions of bovine serum albumin (BSA) were prepared (125, 250, 500, 750, 1000, 1500 and 2000 μg/mL) to build a calibration curve, as specifically indicated in the BCA kit.

#### 4.5.3. ELISA Test

ELISA tests were carried out to define the amounts of three antioxidant enzymes (SOD-1, CAT and GSS). Each microplate was pre-coated with a specific antibody. Of each sample, 100 μL was loaded into the wells and the ELISA plate was covered with a plate sealer for 1 h at 37 °C. Excess samples were discarded and 100 μL of Detection Reagent A was incubated for 1 h at 37 °C. Each well was rinsed with the wash buffer three times and then filled with 100 μL of Detection Reagent B: the samples were incubated again for 1 h at 37 °C and washed five times with the wash buffer. Finally, 90 μL of the substrate solution were added for 15 min at 37 °C. The reaction was stopped with 50 μL of the stop solution, and the absorbance read at 450 nm. A calibration curve was prepared with seven standard solutions according to kit instructions (10, 5, 2.5, 1.25, 0.625, 0.312 and 0.156 ng/mL for SOD-1 and GSS; 4000, 2000, 1000, 500. 250, 125 and 62.5 pg/mL for CAT); a negative control was also set.

### 4.6. Statistical Analysis

Data are reported as mean ± standard deviation (SD). Statistical comparison among three or more groups was conducted using two-way ANOVA followed by Tukey’s post hoc test for multiple comparison to determine significance that was set to *p* < 0.05. For each blood sampling and homogenate sample technical triplicate and quadruplicate were obtained and averaged. Statistical analyses were performed using Prism Graphpad 5 (San Diego, CA, USA).

## 5. Limitations and Conclusions

The beneficial effects of polyphenols, and consequently of propolis, are still a matter of debate due to contradictory results. Our study is the first demonstration that oral uptake of brown propolis is followed by rapid metabolism and by cellular adaptation through the modulation of the concentration of first line antioxidant enzymes. 

This work has limitations and strengths. First, we analyzed the bioavailability of only two representative polyphenols in the sampled propolis, omitting others found at concentrations lower than the limit of detection of the analytical method. Furthermore, we measured the concentration of antioxidant enzymes and not their activity, and limited this to the liver as a tissue of first passage, and not in other tissues. We can only speculate on the exact biochemical mechanism underlying the increased expression of antioxidant enzymes following prolonged administration of propolis. 

On the other side, the major strength of this work is that unlike several works in which both in vitro or in vivo effects of propolis were analyzed through the dosage of a single polyphenolic compound [8,27,28,29,30], our experimental design uses a chemically characterized polyphenolic mixture obtained from brown propolis. With this in mind, we cannot exclude that obtained results may depend on possible synergisms between polyphenols, peculiarly mixed in the propolis sample. 

Future investigations are necessary to more thoroughly reveal the temporal course, qualitative and quantitative adaptations to whole propolis assumption and its effects on cellular redox state. In particular, it would be of great interest to investigate whether a cyclic administration of propolis can represent an effective strategy in maintaining the antioxidant potential in animal models and humans. 

## Figures and Tables

**Table 1:** The peak area for each polyphenol occurring in dry Multi Dinamic Extraction (M.E.D.) extract of brown propolis was identified through the mass spectrum.

Polyphenols	% Area
Quercetin	1.3
Apigenin	1.8
Pinobanksin	1.6
Chrysin	7.5
Pinocembrin	5.0
Galangin	7.8

**Table 2:** Different times at which blood drawings were performed after the acute treatment are listed. In order to minimize animal sufferings, each mouse underwent only two drawings.

Mouse	Time 1	Time 2	Mouse	Time 1	Time 2
1	5′	20′			
2	10′	25′	16	10′	25′
3	15′	30′	17	15′	30′
4	5′	20′	18	30″	45′
5	10′	25′	19	60′	120′
6	15′	30′	20	30″	45′
7	5′	20′	21	60′	120′
8	10′	25′	22	30″	45′
9	15′	30′	23	60′	120′
10	5′	20′	24	30″	45′
11	10′	25′	25	60′	120′
12	15′	30′	26	30″	45′
13	5′	20′	27	60′	120′
14	10′	25′	28	30″	45′
15	15′	25′	29	60′	45′

**Table 3:** The elution method used for the RP-HPLC-PDA-ESI-MSn analysis to determine the polyphenol content in the variety of samples. The eluent A is 0.1% formic acid while eluent B is 100% methanol.

Time (min)	% Eluent A	% Eluent B
0	70	30
10	60	40
30	45	55
50	30	70
55	0	100
60	0	100
65	70	30
70	70	30

## References

[1] Park Y.K., Paredes-Guzman J.F., Aguiar C.L., Alencar S.M., Fujiwara F.Y. (2004). Chemical constituents in *Baccharis dracunculifolia* as the main botanical origin of southeastern Brazilian propolis. J. Agric. Food Chem..

[2] Daugsch A., Moraes C.S., Fort P., Park Y.K. (2008). Brazilian red propolis—Chemical composition and botanical origin. Evid. Based Complement. Altern. Med..

[3] Castaldo S., Capasso F. (2002). Propolis, an old remedy used in modern medicine. Fitoterapia.

[4] Alencar S.M., Oldoni T.L.C., Castro M.L., Cabral I.S.R., Costa-Neto C.M., Cury J.A., Rosalen P.L., Ikegaki M. (2007). Chemical composition and biological activity of a new type of Brazilian propolis: Red propolis. J. Ethnopharmacol..

[5] Marcucci M.C. (1995). Propolis: Chemical composition, biological properties and therapeutic activity. Apidologie.

[6] De Castro S.L. (2001). Propolis: Biological and pharmacological activities. Therapeutic uses of this bee-product. Annu. Rev. Biomed. Sci..

[7] Juliano C., Pala C.L., Cossu M. (2007). Preparation and characterisation of polymeric films containing propolis. J. Drug Deliv. Sci. Technol..

[8] Wang T., Chen L., Wu W., Long Y., Wang R. (2008). Potential cytoprotection: Antioxidant defence by caffeic acid phenethyl ester against free radical-induced damage of lipids, DNA, and proteins. Can. J. Physiol. Pharmacol..

[9] Casaroto A.R., Lara V.S. (2010). Phytomedicines for Candida-associated denture stomatitis. Fitoterapia.

[10] Kurek-Górecka A., Rzepecka-Stojko A., Górecki M., Stojko J., Sosada M., Świerczek-Zięba G. (2013). Structure and antioxidant activity of polyphenols derived from propolis. Molecules.

[11] Chee H.Y. (2002). In vitro evaluation of the antifungal activity of propolis extract on *Cryptococcus neoformans* and *Candida albicans*. Mycobiology.

[12] Banskota A.H., Tezuka Y., Adnyana I.K., Midorikawa K., Matsushige K., Message D., Huertas A.A., Kadota S. (2000). Cytotoxic, hepatoprotective and free radical scavenging effects of propolis from Brazil, Peru, The Netherlands and China. J. Ethnopharmacol..

[13] Zaccaria V., Curti V., Di Lorenzo A., Baldi A., Maccario C., Sommatis S., Mocchi R., Daglia M. (2017). Effect of green and brown propolis extracts on the expression levels of microRNAs, mRNAs and proteins, related to oxidative stress and inflammation. Nutrients.

[14] Porrini M., Riso P. (2008). Factors influencing the bioavailability of antioxidants in foods: A critical appraisal. Nutr. Metab. Cardiovasc. Dis..

[15] D’Archivio M., Filesi C., Varì R., Scazzocchio B., Masella R. (2010). Bioavailability of the polyphenols: Status and controversies. Int. J. Mol. Sci..

[16] Hausen B.M., Wollenweber E., Senff H., Post B. (1987). Propolis allergy: (I). Origin, properties, usage and literature review. Contact Dermat..

[17] Gardana C., Simonetti P., Berti C., Pietta P. (2007). Evaluation of propolis polyphenols absorption in humans by liquid chromatography/tandem mass spectrometry. Rapid. Commun. Mass Spectrom..

[18] Nabavi S.F., Braidy N., Habtemariam S., Orhan I.E., Daglia M., Manayi A., Gortzi O., Nabavi S.M. (2015). Neuroprotective effects of chrysin: From chemistry to medicine. Neurochem. Int..

[19] Konishi Y., Hitomi Y., Yoshida M., Yoshioka E. (2015). Absorption and bioavailability of artepillin c in rats after oral administration. J. Agric. Food Chem..

[20] Konishi Y., Hitomi Y., Yoshida M., Yoshioka E. (2015). Pharmacokinetic study of caffeic and rosmarinic acids in rats after oral administration. J. Agric. Food Chem..

[21] Konishi Y., Kobayashi S. (2004). Microbial metabolites of ingested caffeic acid are absorbed by the monocarboxylic acid transporter (MCT) in intestinal Caco-2 cell monolayers. J. Agric. Food Chem..

[22] Konishi Y., Hitomi Y., Yoshioka E. (2004). Intestinal absorption of *p* -coumaric and gallic acids in rats after oral administration. J. Agric. Food Chem..

[23] Zhao Z., Egashira Y., Sanada H. (2004). Ferulic acid is quickly absorbed from rat stomach as the free form and then conjugated mainly in liver. J. Nutr..

[24] Lahouel M., Boulkour S., Segueni N., Fillastre J.P. (2004). The flavonoids effect against vinblastine, cyclophosphamide and paracetamol toxicity by inhibition of lipid-peroxydation and increasing liver glutathione concentration. Pathol. Biol. (Paris).

[25] De Barros M.P., Lemos M., Maistro E.L., Leite M.F., Sousa J.P.B., Bastos J.K., de Andrade S.F. (2008). Evaluation of antiulcer activity of the main phenolic acids found in Brazilian green propolis. J. Ethnopharmacol..

[26] Liu C.F., Lin C.C., Lin M.H., Lin Y.S., Lin S.C. (2002). Cytoprotection by propolis ethanol extract of acute absolute ethanol-induced gastric mucosal lesions. Am. J. Chin. Med..

[27] Jasprica I., Mornar A., Debeljak Ž., Smolčić-Bubalo A., Medić-Šarić M., Mayer L., Romić Ž., Bućan K., Balog T., Sobočanec S., Šverko V. (2007). In vivo study of propolis supplementation effects on antioxidative status and red blood cells. J. Ethnopharmacol..

[28] Benz C.C., Yau C. (2008). Ageing, oxidative stress and cancer: Paradigms in parallax. Nat. Rev. Cancer.

[29] Favier A. (1997). Oxidative stress: Value of its demonstration in medical biology and problems posed by the choice of a marker. Ann. Biol. Clin. (Paris).

[30] Zamora-Ros R., Knaze V., Rothwell J.A., Hémon B., Moskal A., Overvad K., Tjønneland A., Kyrø C., Fagherazzi G., Boutron-Ruault M.C. (2016). Dietary polyphenol intake in europe: The european prospective investigation into cancer and nutrition (EPIC) study. Eur. J. Nutr..

[31] Chen B., Lu Y., Chen Y., Cheng J. (2015). The role of Nrf2 in oxidative stress-induced endothelial injuries. J. Endocrinol..

[32] Zhao L., Pu L., Wei J., Li J., Wu J., Xin Z., Gao W., Guo C. (2016). Brazilian green propolis improves antioxidant function in patients with type 2 diabetes mellitus. Int. J. Environ. Res. Public Health.

[33] Williams R.J., Spencer J.P., Rice-Evans C. (2004). Flavonoids: Antioxidants or signalling molecules?. Free Radic. Biol. Med..

[34] Zelko I.N., Mariani T.J., Folz R.J. (2002). Superoxide dismutase multigene family: A comparison of the CuZn-SOD (SOD1), Mn-SOD (SOD2), and EC-SOD (SOD3) gene structures, evolution, and expression. Free Radic. Biol. Med..

[35] Cui-ping Z., Shuai H., Wen-ting W., Shun P., Xiao-ge S., Ya-jing L., Fu-liang H. (2014). Development of high-performance liquid chromatographic for quality and authenticity control of Chinese propolis. J. Food Sci..

[36] Sharma A., Fish B.L., Moulder J.E., Medhora M., Baker J.E., Mader M., Cohen E.P. (2014). Safety and blood sample volume and quality of a refined retro-orbital bleeding technique in rats using a lateral approach. Lab Anim..

